# Novel Vectors of Malaria Parasite in the Western Highlands of Kenya

**DOI:** 10.3201/eid1809.120283

**Published:** 2012-09

**Authors:** Jennifer Stevenson, Brandyce St. Laurent, Neil F. Lobo, Mary K. Cooke, Samuel C. Kahindi, Robin M. Oriango, Ralph E. Harbach, Jonathan Cox, Chris Drakeley

**Affiliations:** London School of Hygiene and Tropical Medicine, London, UK (J. Stevenson, M.K. Cooke, J. Cox, C. Drakeley);; University of Notre Dame, Notre Dame, Indiana, USA (B. St. Laurent, N.F. Lobo);; US Centers for Disease Control and Prevention, Kisumu, Kenya (S.C. Kahindi, R.M. Oriango);; and Natural History Museum, London (R.E. Harbach)

**Keywords:** malaria, vector, parasite, eukaryotic protist, mosquito, Kenya, anopheliene mosquitoes, Anopheles, vector-borne infections

## Abstract

The main method of malaria control is based on a simple premise: avoid mosquito bites by killing the mosquitoes. This concept relies on spraying insecticides indoors and sleeping under insecticide-treated bed nets because it is assumed that malaria mosquitoes spend most of their time indoors and feed at night. That is, until now. A recent study has identified new species of mosquitoes that prefer to be outdoors and to feed earlier in the evening. These behavior patterns could render current control practices ineffective. New malaria control methods need to be developed according to the specific behavior of all the different vectors.

**To the Editor:** The primary malaria control techniques, indoor application of residual insecticides and insecticide-treated bed nets, are used on the basis of previously assumed key characteristics of behaviors of vectors of malaria parasites, i.e., resting and feeding indoors (*1*). Any deviation from the typical activities of a species related to exophagy (feeding outdoors) and exophily (living and resting indoors) (*2*) or to population replacement, followed by increased outdoor biting or resting (*3*), may undermine malaria control efforts. Identification of mosquitoes that transmit malaria parasites has, for the most part, relied on the use of outdated morphologic keys (*4*,*5*) and, more recently, species-diagnostic PCR (*6*). Cryptic species or subpopulations that exhibit divergent behaviors (*7*) may be responsible for maintaining malaria parasite transmission, and without adequate discriminatory techniques, these vectors may be misidentified and their key behavioral differences overlooked.

We evaluated indoor and outdoor trapping methods for anopheline mosquitoes in Bigege village, in Kisii Central District in the highlands of western Kenya, which are prone to periodic malaria epidemics. During May–August 2010, we captured 422 female *Anopheles* spp. mosquitoes, primarily from indoor and outdoor light traps. Of these, we identified 161 (38.2%) as species previously described as vectors in the area (*An. gambiae* sensu latu, *An. funestus* s.l., or *An. coustani* [*8*]) by using the standard morphologic key for sub-Saharan species (*4*). We identified another 52 (12.3%) as species not associated with malaria parasite transmission (*1*), but 209 (49.5%) could not be definitively identified. We extracted DNA from 418 mosquitoes and analyzed it for sibling species of the *An. gambiae* complex by using a diagnostic PCR (*6*). Of the 418 DNA samples tested, 80 (19.1%) were identified as *An. arabiensis*; 2 specimens were identified as *An. gambiae* s.s. (0.4%) but the remaining 336 (80.3%) could not be identified by PCR because no amplification product was observed.

To identify these specimens further, we performed molecular characterization by sequencing the ribosomal second internal transcribed spacer (ITS2) and the mitochondrial CO1 loci. Of the 422 female *Anopheles* mosquito specimens, we sequenced DNA from 348, of which 74 (21.3%), 33 (9.5%), and 25 (7.2%) corresponded to GenBank sequences of *An. arabiensis*, *An. coustani*, and *An. funestus* mosquitoes, respectively. However, 216 (62.1%) could not be matched (<90% identity) to any of the 224 ITS2 or 164 CO1 published sequences of anopheline vectors or nonvectors. These 216 specimens could be grouped into several separate clades, distinct from known vectors in the area (Figure). Specimens were grouped by ITS2 sequence. These groups were ranked by abundance and arbitrarily named species A-J. Of the 348 sequenced DNA specimens, the most abundant group having identical but novel ITS2 and CO1 sequences (species A, n = 147, 42.2%) could not be matched definitively to a single species by using the morphologic key. The mosquitoes in this group were most frequently caught outdoors (132, 89.8%). For 64 of a total of 192 traps, collections were made every 2 hours between 6:30 pm and 6:30 am for 64 nights. Of 30 specimens of species A from these collections, 22 (73.3%) were caught outdoors before 10:30 pm. Data we have collected on human sleeping patterns from this area suggest that a significant proportion of the population is still outdoors before 10:30 pm and therefore exposed to these vectors.

**Figure:**
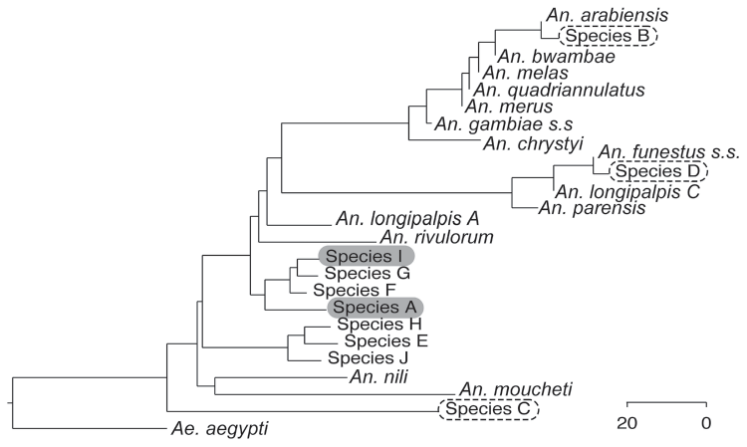
Phylogenetic tree of sequence group consensuses with National Center of Biotechnology Information reference sequences for *Anopheles* spp. mosquitoes caught in 2010 in Kisii District, Nyanza Highlands, western Kenya. Sequence groups of caught specimens arbitrarily named species A to J are ranked by abundance. Gray highlighting indicates study samples with sporozoites; dashed circles around text indicate study samples that match known African vectors. Scale bar represents nucleotide substitutions per 100 residues.

Five of 293 mosquitoes tested had ELISA results positive for *Plasmodium falciparum* sporozoites. All 5 had no previously published ITS2 or CO1 sequences, nor could they be identified by morphologic features. All were collected outdoors. Four of the 5 were in the sequence A group (Technical Appendix), and 1 belonged to species I (Figure). The sporozoite rate of 3% in species A was similar to that observed for other predominant anopheline vectors in the area (*8*).

Since the publication of the most widely available morphologic key (*4*), 15 new anopheline species have been discovered, for which test results for 1, *An. ovengensis* from Cameroon, were confirmed to be positive for sporozoites (*9*). The unidentified mosquitoes in the current study did not match the morphologic descriptions of any of the more recently identified species. These results demonstrate the presence of outdoor-active, early-biting potential malaria parasite vectors not previously described in western Kenya. The outdoor activity of these mosquitoes could lead to the failure of current indoor-based interventions to control this species, and this species could therefore contribute to malaria parasite transmission in the area. These findings highlight the value of the use of characteristics of local *Anopheles* spp. populations, including their behavior, based on morphologic features and DNA analysis, to accurately determine whether the species is contributing to malaria parasite transmission. This knowledge is essential for implementation of appropriate, and therefore successful, malaria control interventions.

Technical Appendix Genetic and morphological identification of female *Anopheles* spp. mosquitoes caught during 2010 in Kisii District, Nyanza Highlands, western Kenya.
