# Supplementary material for: Novel Vectors of Malaria Parasite in the Western Highlands of Kenya
Source: Emerg Infect Dis. 2012 Sep;18(9):1547–9. doi: 10.3201/eid1809.120283 (PMC3437730; doi:10.3201/eid1809.120283)
Supplement: Technical Appendix — Genetic and morphological identification of female Anopheles spp. mosquitoes caught during 2010 in Kisii District, Nyanza Highlands, western Kenya. [file 12-0283-Techapp-s1.pdf]

Article DOI: http://dx.doi.org/10.3201/eid1809.120283

Novel Vectors of Malaria Parasite in the Western Highlands of Kenya

Technical Appendix

Technical Appendix Table. Genetic and morphological identification of female *Anopheles* spp. mosquitoes caught during 2010 in Kisii District, Nyanza Highlands, western Kenya*

Sequence group†	ITS2 sequence homology	CO1sequence homology	Closest species from morphological key (*4*)	No. female (% total catch)	No. tested for *P. falciparum* sporozoites	No. positive for sporozoites (% sequence group)
A	‡	‡	*An. funestus/An. demeilloni*	147 (42.2)	129	4 (3.1)
B	*An. arabiensis*	*An. arabiensis*	*An. gambiae*	74 (21.3)	61	0
C	‡	*An. coustani*	*An. coustani*	33 (9.5)	28	0
D	*An. funestus*	*An. funestus*	*An. funestus*	25 (7.2)	22	0
E	‡	‡	*An. maculipalpis*	22 (6.3)	18	0
F	‡	‡	Mixed	12 (3.4)	8	0
G	‡	‡	Mixed	13 (3.7)	9	0
H	‡	‡	Mixed	9 (2.6)	6	0
I	‡	‡	*An. harperi*	8 (2.3)	7	1 (14.3)
J	‡	‡	Mixed	5 (1.4)	4	0
Total	NA	NA	NA	348	292	5 (1.7)

*ITS2, ribosomal second internal transcribed spacer; CO1, mitochondrial cytochrome c oxidase subunit 1; †Sequence groups of caught specimens arbitrarily named species A–J are ranked by abundance. NA, not applicable; ‡, no published sequences found with at least 90% homology to those of specimens.
